# Early detection of influenza outbreaks using the DC Department of Health's syndromic surveillance system

**DOI:** 10.1186/1471-2458-9-483

**Published:** 2009-12-22

**Authors:** Beth Ann Griffin, Arvind K Jain, John Davies-Cole, Chevelle Glymph, Garret Lum, Samuel C Washington, Michael A Stoto

**Affiliations:** 1 RAND Corporation, Center for Domestic and International Health Security, 1200 Hayes Street, Arlington, VA 22202-5050 USA; 2 District of Columbia Department of Health, 825 North Capitol Street NE, Washington, DC 20002 USA; 3 Georgetown University Department of Health Systems Administration, 3700 Reservoir Rd. NW, Washington, DC 20007 USA

## Abstract

**Background:**

Since 2001, the District of Columbia Department of Health has been using an emergency room syndromic surveillance system to identify possible disease outbreaks. Data are received from a number of local hospital emergency rooms and analyzed daily using a variety of statistical detection algorithms. The aims of this paper are to characterize the performance of these statistical detection algorithms in rigorous yet practical terms in order to identify the optimal parameters for each and to compare the ability of two syndrome definition criteria and data from a children's hospital versus vs. other hospitals to determine the onset of seasonal influenza.

**Methods:**

We first used a fine-tuning approach to improve the sensitivity of each algorithm to detecting simulated outbreaks and to identifying previously known outbreaks. Subsequently, using the fine-tuned algorithms, we examined (i) the ability of unspecified infection and respiratory syndrome categories to detect the start of the flu season and (ii) how well data from Children's National Medical Center (CNMC) did versus all the other hospitals when using unspecified infection, respiratory, and both categories together.

**Results:**

Simulation studies using the data showed that over a range of situations, the multivariate CUSUM algorithm performed more effectively than the other algorithms tested. In addition, the parameters that yielded optimal performance varied for each algorithm, especially with the number of cases in the data stream. In terms of detecting the onset of seasonal influenza, only "unspecified infection," especially the counts from CNMC, clearly delineated influenza outbreaks out of the eight available syndromic classifications. In three of five years, CNMC consistently flags earlier (from 2 days up to 2 weeks earlier) than a multivariate analysis of all other DC hospitals.

**Conclusions:**

When practitioners apply statistical detection algorithms to their own data, fine tuning of parameters is necessary to improve overall sensitivity. With fined tuned algorithms, our results suggest that emergency room based syndromic surveillance focusing on unspecified infection cases in children is an effective way to determine the beginning of the influenza outbreak and could serve as a trigger for more intensive surveillance efforts and initiate infection control measures in the community.

## Background

In a typical year, influenza results in 36,000 or more deaths and more than 200,000 hospitalizations in the United States alone. In addition to this human toll, influenza is annually responsible for a total cost of over $10 billion in the United States. A pandemic, or worldwide outbreak of a new influenza virus such as the AH1N1 virus that emerged in 2009, could dwarf this impact by overwhelming our health and medical capabilities, potentially resulting in hundreds of thousands of deaths, millions of hospitalizations, and hundreds of billions of dollars in direct and indirect costs [1].

Epidemiological characteristics of both seasonal and pandemic influenza suggest that syndromic surveillance systems are likely to make an important contribution beyond the capabilities of existing surveillance systems, and thus enable a more effective public health response to influenza outbreaks in a particular area. Indeed, a number of studies have demonstrated the potential that syndromic surveillance of influenza-like illness (ILI) offers, and such systems are now common at the national, state, and local levels [2]. Brownstein and colleagues [3] show that children and infants presenting to pediatric emergency departments (ED) with respiratory syndromes represent an early indicator of impending influenza morbidity and mortality, sometimes with as much as a three week lead on EDs serving adults. Lemay and colleagues [4] have found similar results in Ottawa, Canada. Using data from New York City, Lu and colleagues [5] have shown that monitoring both outpatient and ED data can enhance detection of ILI outbreaks. Lau and colleagues [6], Zheng and colleagues [7], and Cooper and colleagues [8] have found similar results in Hong Kong (China), New South Wales (Australia), and the United Kingdom respectively. Olson and colleagues [9] note that age-stratified analyses of ED visits for fever and respiratory complaints offer the potential earlier warning of the arrival of epidemic influenza (compared to non-stratified analyses) because they allow for detection of the characteristic age-shift of pandemic influenza. Olson and colleagues also identified the impact of respiratory syncytial virus (RSV) in children earlier than the flu season most years [9]. More recently, Polgreen and colleagues [10] and Ginsberg and colleagues [11] have suggested that data describing internet searches for flu symptoms could also be useful in detecting influenza outbreaks.

Statistical detection algorithms play a critical role in any syndromic surveillance system, yet at times they are applied without careful considerations of their statistical properties and performance. For example, many syndromic surveillance systems utilize "packaged" statistical detection algorithms such as the Centers for Disease Control and Prevention's (CDC) Early Aberrations Reporting System (EARS) [12] with little control over the parameters values used in those algorithms. However, selection of parameter values for an algorithm has a large impact on the statistical performance of the algorithm. In general, the performance of a statistical detection algorithm is characterized by a tradeoff among three factors: sensitivity, false positive rate, and timeliness. Sensitivity represents an algorithm's ability to flag when an outbreak is really happening. The false positive rate represents the likelihood it alarms incorrectly, when an outbreak is not occurring, and timeliness refers to an algorithm's ability to flag as early as possible when an outbreak is occurring.

The goals of this paper were two-fold. First, we studied the performance of three commonly used statistical detection algorithms against detecting simulated outbreaks and previously "known" outbreaks detected in data from the District of Columbia's (DC) Department of Health's Emergency Room Syndromic Surveillance System (ERSSS) [13]. The three algorithms included a standard CUSUM, a version of the CUSUM based on deviations from an exponentially weighted moving average, and the multivariate CUSUM [13]. Our primary aim in this analysis was to fine-tune the key parameters in each detection algorithm to ensure optimal performance in terms of reasonable trade-offs between sensitivity, timeliness, and the false positive rate of the algorithms. Additionally, we compared the three fine-tuned versions of the algorithms to determine which algorithm offered optimal performance for public health practice and used the improved detection algorithms to characterize the DC ERSSS's performance with regard to determining the onset of seasonal influenza outbreaks.

Second, we aimed to assess the performance of the DC ERSSS. To do so, we compared a number of different 'candidate' syndromic surveillance systems within the system to determine which offered the greatest benefit in detecting the beginning of influenza outbreaks. Specifically, we examine the use of data from DC's Children's National Medical Center (CNMC) as a sentinel syndromic surveillance system and the use of different symptom groups both individually and together to determine which conditions are strongly correlated with the beginning of influenza outbreaks. Some studies have suggested that children's symptoms are an especially sensitive indicator of the start of seasonal influenza outbreaks. Children, and the elderly, are likely to be the first victims of influenza due to their weaker immune systems. Such results have been seen in the analysis of ER surveillance data from both Boston [14] and New York [9]. Given such high sensitivity to influenza, it is important to study more comprehensively the possible benefits that could be gleamed from a syndromic surveillance system which focuses on data from hospitals whose primary patients are children as we do here.

## Methods

In the proceeding sections, we regard a flu outbreak as a period characterized by a sudden increase in the number of people with flu-like symptoms in a given location. Since flu outbreaks in the United States usually occur from December through April, we defined the "flu season" to be December 1 through April 30 of the subsequent calendar year and the "non-flu season" to be the remainder of the year.

### Data collection

Data for this analysis came from DC's ERSSS. In DC, emergency department logs from nine hospitals are sent on a daily basis to the health department, where health department staff code them on the basis of chief complaint, recording the number of patients in each of the following mutually exclusive syndromic categories: death, sepsis, rash, respiratory illness, gastrointestinal illness, unspecified infection, neurological illness, and other complaints [15]. Coding is done hierarchically, in the order given, so patients with two or more complaints will be assigned the first one on the list. The data span the calendar dates of September 11, 2001 to June 19, 2006, i.e. 1,743 days. We used only part of the data, representing the time between September 11, 2001 and May 17, 2004 (the period covered by our previous research) as a test sample to determine the parameter values in our fine-tuning analysis. The remaining data were set aside until this was accomplished and the complete data set used to validate our choices, as described in more detail below.

With the exception of some of the first days of the program, daily counts that were missing were imputed using the imputation strategy described in the appendix. After imputing the missing counts in the data, we standardized the daily counts for each condition and hospital by dividing the daily count by the mean number of cases in the non-flu seasons.

### Fine-tuning approach

To fine-tune the three algorithms defined below, we used a two-pronged approach that aims to characterize the performance of the algorithms in rigorous, yet practical terms. First, we utilized a simulation study to estimate receiver operating characteristic (ROC) curves and determine which parameter values provided an optimal trade-off between false positive rates and sensitivity of an algorithm. Second, we used "known" outbreaks in the DC Department of Health data to assess the timeliness and sensitivity of an algorithm when faced with detecting actual (non-simulated) outbreaks. Data available for the initial fine-tuning analysis spanned the calendar dates from September 12, 2001 to May 17, 2004, i.e. 980 days

To perform the simulation study, we first created 970 datasets from the DC data with simulated outbreaks as follows. Linearly increasing outbreaks were inserted into each dataset such that x extra cases were inserted on day one of the outbreak, 2× on day two, and 3× on day three. The datasets each had a different start date for the simulated 3-day outbreak, that is, the first dataset's outbreak began on September 12, 2001, the second's on September 13, 2001, and the 970th data set's on May 8, 2004. Since we use standardized daily counts, x was set equal to values between zero and one where x = 0.50 would correspond to inserting multiples of half the average number of daily cases in the non-flu seasons into the data.

After creating the simulated datasets, we applied the algorithms for a given set of parameter values and false positive rate (between 0.001 and 0.05) to each one. We then computed the sensitivity of the algorithm to detect the simulated outbreak by day three of the outbreak in the non-flu seasons over all the simulated data sets. It was of primary interest to understand how well the algorithms did at detecting a simulated outbreak against a "normal" background level of disease activity. No known outbreaks occurred during the "normal" background periods. For this reason we did not use data from the flu seasons, December 1 to April 30, which likely contain actual outbreaks in addition to the simulated ones, yielding biased sensitivity rates. Thus, we computed the sensitivity of the algorithm using the following formula:

Finally, we plotted the sensitivity of the algorithm to flag by day three in the non-flu seasons by the false positive rate and computed the area under the sensitivity curve (see below). The set of parameters of the algorithm that gave the curve with the maximum area was considered to be the optimal set of parameter values in the simulation study since it gave the best balance between sensitivity and false positive rates [16].

After selecting a small number of candidate values that performed well under the ROC curve approach, we proceeded to the second part of our fine-tuning analysis. In the second part of our analysis, we assessed how well the selected candidate values did at detecting previously "known" outbreaks in the DC Department of Health data set. Specifically, we assessed how well the algorithms did at flagging the beginnings of the flu outbreaks in 2002 and 2004 and at flagging gastrointestinal outbreaks that occurred in three hospitals during the winter of 2003.

We selected data from three hospitals for our fine-tuning analysis based on the number of emergency department admissions. Throughout this paper, we refer to these as hospitals S (small), M (medium), and L (large). For each, we used data for two conditions: unspecified infection and gastrointestinal.

**Figure 1:**
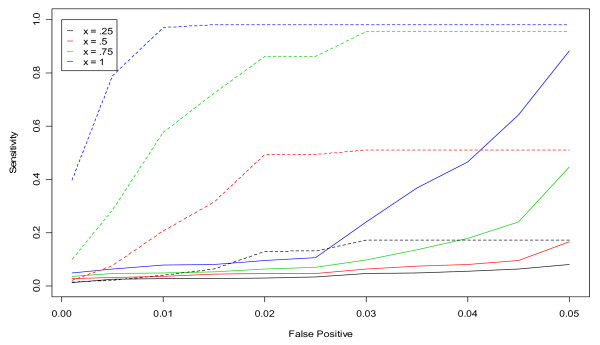
**ROC curves for detection of a simulated outbreak by day 3 in the non-flu seasons in hospital S for unspecified infection using the CUSUM algorithm**. Dashed and solid lines correspond to CUSUM algorithms with *k *= 1.5 and *k *= 0.5, respectively.

**Figure 2:**
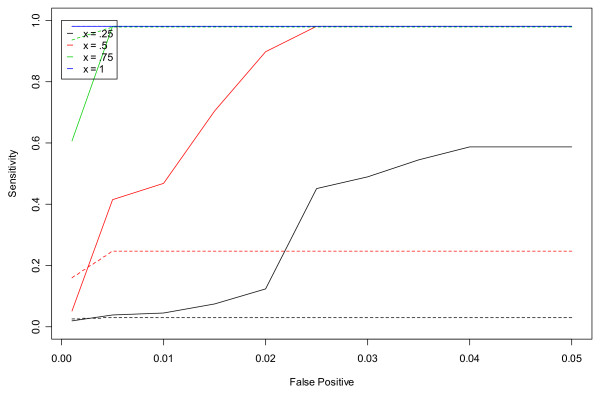
**ROC curves for detection of a simulated outbreak by day 3 in the non-flu seasons in hospital L for unspecified infection using the CUSUM algorithm**. Dashed and solid lines correspond to CUSUM algorithms with *k *= 1.5 and *k *= 0.5, respectively.

The results of our fine-tuning analysis are presented primarily in two different graphical formats. The results of the simulation studies are presented in terms of ROC curves, as in Figures 1 and 2. For this format, the horizontal axis represents the false positive rate and the vertical axis represents the sensitivity rate. The curves display the sensitivity rate on day three of the simulated outbreak in the non-flu season that is achieved for each pre-determined false positive rate. (The non-flu season is used under the assumption that there are no actual outbreaks during this period.) The different curves correspond to different outbreak sizes or parameter values.

**Figure 3:**
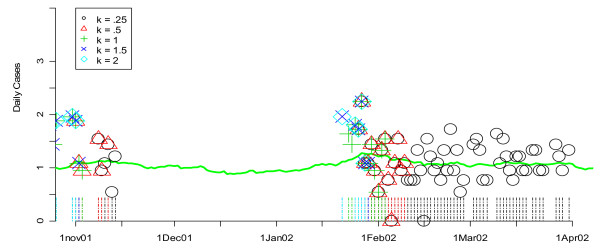
**Daily flags from the CUSUM algorithm for *k *= 0.25, 0.50, 1, 1.5, and 2 applied to hospital S's unspecified infection daily counts during the winter of 2002**. Green lines show the smoothed values for unspecified infection daily counts from the given hospital. Flagging times are also represented along the time axis with vertical lines that are color coded to the symbol. Note that the vertical axis is on a square root scale because of its variance stabilizing properties.

**Figure 4:**
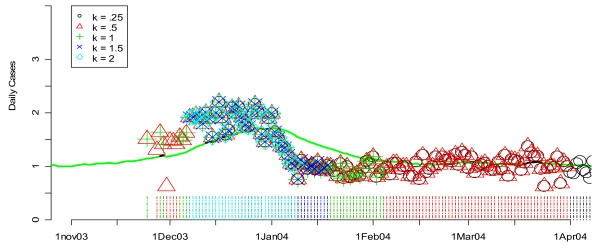
**Daily flags from the CUSUM algorithm for *k *= 0.25, 0.50, 1, 1.5, and 2 applied to hospital L's unspecified infection daily counts during the winter of 2004**. Green lines show the smoothed values for unspecified infection daily counts from the given hospital. Flagging times are also represented along the time axis with vertical lines that are color coded to the symbol. Note that the vertical axis is on a square root scale because of its variance stabilizing properties.

The application of the detection algorithms to actual data in comparison with known outbreaks is displayed in a different format, as exemplified by Figures 3 and 4. In this form, smoothed values for one or more data streams (unspecified infection cases in hospital S in Figure 3 and hospital L in Figure 4) are presented in terms of a curve. The flagging of the detection algorithms is represented by symbols plotted according to the day they flagged on the horizontal axis and the value of the test statistic (e.g. CUSUM) on that date on the vertical axis. The flagging times are also represented along the time axis with vertical lines that are color coded to the symbol. Note that the vertical axis is on a square root scale because of its variance stabilizing properties. "Winter" is used in these graphs and throughout the remainder of the paper as a shorthand for November 1 of the previous year to April 1 of the year displayed or discussed. This definition, which differs from our definition of the "flu season," was chosen simply to ensure that the figures focus on the part of the data where flu outbreaks actually began during the study period.

### Statistical detection algorithms

We utilize three detection algorithms in this study. The first, the CUSUM (for "cumulative summation") algorithm, monitors the daily statistic *S*_*i *_which is defined by the recursive formula

where *X*_*i *_denotes the observed daily count on day i, *μ *denotes the overall mean daily count estimated from the data, and k is an off-set parameter set by the user [17,18]. This statistic cumulates positive deviations from the average in order to detect small but persistent increases in cases. The CUSUM algorithm alarms or flags at time  = inf{*i *: *S*_*i *_>*h*} where h is computed empirically to guarantee a fixed false positive rate (specified by the user) in the non-flu seasons. In our analysis, we focus on fine-tuning the user-specified value of k. The parameter *k *is a result of the theoretical derivation of the univariate CUSUM as a sequential likelihood ratio test for a shift *δ *in the mean parameter of a normal distribution. Under standard assumptions, *k *is usually set to *δ*/2, for a one-time shift in level that must be detected quickly. In syndromic surveillance however, we typically expect outbreaks that increase in size over time, so the standard theory does not determine an optimal *k*. Instead, *k *must be determined empirically using methods such as those described below. The theory does, however, suggest that choice of *k *matters a great deal. If *k *is too small, the algorithm will detect every fluctuation in the data while if it is too large, the algorithm will not detect anything at all.

The CUSUM based on deviations from an exponentially weighted moving average, which we refer to throughout as EXPO, adds one additional step to the CUSUM algorithm described above [13]. First, the EXPO algorithm predicts the daily counts, *X*_*i*_, using an exponentially weighted moving average. Specifically, it defines

where 0 ≤ *λ *≤ 1 is a user-specified parameter and represents the degree of smoothing that is to be done in the data (i.e. smaller values correspond to more smoothing). The algorithm then monitors the differences between the actual and predicted counts using the statistic *S*_*i *_which is defined by the following recursive formula

As with CUSUM, the EXPO algorithm flags at time  = inf{*i *: *S*_*i *_>*h*} where *h *is computed empirically to guarantee a fixed false positive rate (user-specified) in the non-flu seasons. In contrast to CUSUM, EXPO should allow for more sensitive detection when outbreaks appear against a linearly increasing background pattern. With EXPO, two parameters must be fine-tuned: *k *and *λ*. Both parameters are supplied by users in this algorithm and their selected values will have a large impact on the statistical performance of the algorithm.

Lastly, we worked to fine-tune the multivariate CUSUM algorithm, which we refer to as MV CUSUM. The MV CUSUM was developed for monitoring multiple streams of data on a daily basis (e.g., streams of data from more than one hospital or streams of data from more than one condition within a hospital) [19] and can offer greater utility when an outbreak is likely to influence the daily counts of more than one symptom group. It follows the same logic as the standard CUSUM except that now daily counts are represented by a vector ***X***_i _whose dimension is *p × 1 *where *p *represents the number of streams being analyzed together. We define

and ***S***_i _= **0 **if *C*_*i *_≤ *k *where

and ***Σ***^-1 ^is the estimated variance-covariance matrix for the *p *streams of data being analyzed using only daily counts from the non-flu seasons. The MV CUSUM algorithm flags at time  = inf{*i *: *C*_*i *_>*h*}. For the MV CUSUM algorithm, *k *is the parameter of interest for fine-tuning purposes. It is user-specified and its value will impact the statistical performance of the algorithm.

### Analysis of DC influenza data

In order to evaluate the ERSSS data's ability to determine the beginning of the seasonal influenza outbreak in DC, we selected a number of 'candidate' syndromic surveillance systems within the system and compared how well each 'candidate' system did at flagging the beginning of the flu season. Additionally, we compared each one to CDC's sentinel physician data, which is based on a network of physicians reporting each week on the proportion of cases they have seen that have influenza-like illness (ILI). Our 'candidate' systems included both univariate and multivariate versions. Initially, we examined how well each of the eight syndrome categories did alone at flagging the beginning of the flu season. Then, based on the initial performance, we focused in on the ability of unspecified infection and respiratory categories to detect the start of the flu season both within a single hospital and taken together across hospitals.

Finally, we examined how well CNMC did versus all the others when using just unspecified infection, just respiratory, and both categories together. For the CDC's sentinel physician data, data is available only on a national and regional basis, so we choose to use the South Atlantic region, which includes the District of Columbia (CDC, various years) in our comparisons. Also, all the comparisons in the influenza analysis used fine-tuned versions of the algorithms based on the results from the fine-tuning analysis.

## Results

### Fine-tuning Analysis

As described above, we began our study by fine-tuning the *k *parameter found in the standard, univariate CUSUM algorithm. We studied the performance of a large range of *k *values beginning with the performance of the univariate CUSUM for *k *= 2, 2.5, 3,..., 6. However, these values performed very poorly. Thus, we closely examined the performance of values of *k *between 0.25 and 2. In general, we found that the following rule of thumb worked well for applying the univariate CUSUM: If a hospital has a mean non-flu season daily rate of less than five cases per day, *k *= 1.5 is optimal for standardized data. If a hospital has a mean non-flu season daily rate of greater than five cases per day, *k *= 0.5 is optimal. Thus, according to Table 1 which shows the mean daily number of cases in the non-flu seasons for each hospital and condition, *k = *1.5 was optimal for hospitals S and M for unspecified infection and *k *= 0.5 was optimal for all other cases.

**Table 1:** Mean number of daily cases in the non-flu seasons for hospitals S, M, and L by unspecified infection and gastrointestinal symptoms.

	Hospital S	Hospital M	Hospital L
Unspecified Infection	3.5	4.8	28.1

Gastrointestinal	9.0	21.9	14.7

Figures 1 and 2 show the results from the ROC curve analysis for hospitals S and L and unspecified infection. Dashed lines represent *k *= 1.5 and solid lines represent *k *= 0.5. The different colored lines represent different outbreak sizes from x = 0.25 to x = 1. As expected, as x increases, the sensitivity of the CUSUM algorithm for both values of *k *increases. As the outbreak size increases, the outbreak is easier to detect for a given false positive rate. In Figure 1 (Hospital S), the dashed lines corresponding to *k *= 1.5 clearly dominate over their partner solid line for each value of x. Conversely, in Figure 2 (Hospital L) which is a larger hospital and has mean daily rate of cases in the non-flu seasons for unspecified infection equal to 28.11, the solid lines corresponding to *k *= 0.5 clearly dominate over the dashed lines of *k *= 1.5 for each x.

Figures 3 and 4 show the performance of the CUSUM algorithm for *k *= 0.25, 0.50, 1.00, 1.50, 2.00 for detecting previously "known" flu outbreaks in hospital S in the winter of 2002 and hospital L in the winter of 2004. Again, for hospital S (Figure 3) which has a small mean daily rate of cases in the non-flu seasons (e.g. 3.48), larger values of *k *(e.g *k *= 1.5 and 2) do the best at indicating the beginning of the flu outbreaks by flagging the earliest. Conversely for hospital L (Figure 4), smaller value of *k *(e.g. *k *= 0.5 and 1) were the first to flag the beginning of the flu outbreak in 2004. Taken together, the results from Figures 1 through 4 suggest that *k *= 1.5 is optimal for hospitals with mean daily non-flu season counts of less than 5 and *k *= 0.5 is optimal for hospitals with mean daily non-flu season counts of greater than 5.

**Figure 5:**
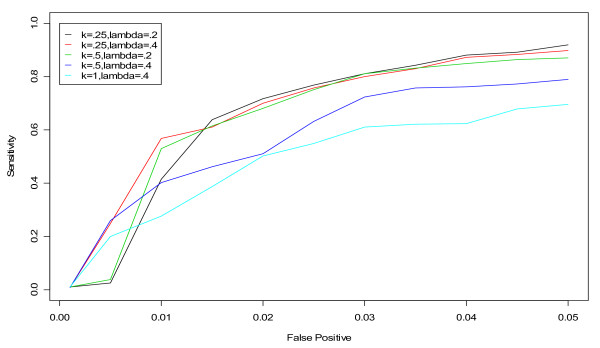
**ROC curves for detection of a simulated outbreak by day 3 in the non-flu seasons in hospital M for unspecified infection using various values of (k, *λ*) in the EXPO algorithm**.

**Figure 6:**
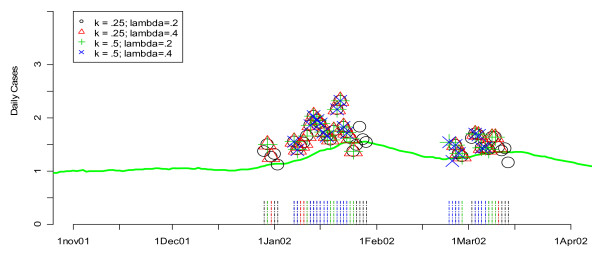
**Flags from the EXPO algorithm for various values of (k, *λ*) applied to Hospital L's unspecified infection daily counts during the winter of 2002**. Green lines show the smoothed values for unspecified infection daily counts from the given hospital. Flagging times are also represented along the time axis with vertical lines that are color coded to the symbol. Note that the vertical axis is on a square root scale because of its variance stabilizing properties.

Repeating the steps above, we began fine-tuning the *k *and *λ *parameters of the EXPO algorithm using the ROC curve approach. Searching for optimal values of (*k*, *λ*) involved an iterative process of first fine-tuning *λ *for *k *= 0.5 and 1.5 and then fixing *λ *between 0.1 and 0.5 and varying *k*. This process was repeated until optimal performance was detected for values of *k *between 0.25 and 0.5 and values of *λ *between 0.2 and 0.4. Interestingly, we found that the same set of values for (*k*, *λ*) did well for all hospitals and conditions. However, we also found that there was far less of a distinction between the groups of selected candidate values for the EXPO algorithm than we found for the selected candidate values in the CUSUM algorithm (Figure 5). The top two sets of values for (*k*, *λ*) were (0.25,0.20) and (*k*, *λ*) = (0.25,0.40) with areas under of the curve of 0.0484 and 0.0485, respectively. In terms of detecting a previously "known" flu outbreak, (*k*, *λ*) = (0.25, 0.20) only flags the beginning of the flu outbreak a couple days before (0.25, 0.40) for hospital L in the winter of 2002 (Figure 6). Based on our findings for all three hospitals and both conditions, we recommend using (*k*, *λ*) = (0.25, 0.20) for the D.C. data since it outperforms (*k*, *λ*) = (0.25, 0.40) even if slightly.

**Figure 7:**
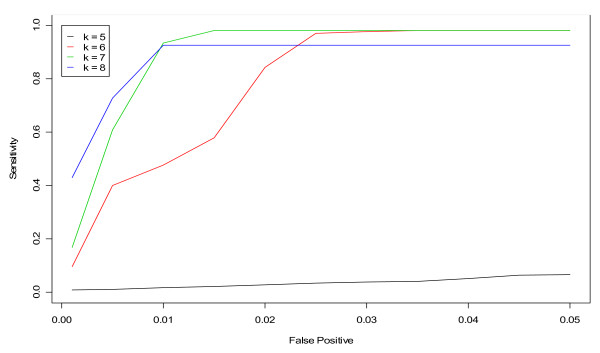
**ROC curves for detection of a simulated outbreak by day 3 in the non-flu seasons using various values of k in the MV CUSUM and applying the algorithm to three streams of unspecified infection data from hospitals S, M, and L**.

**Figure 8:**
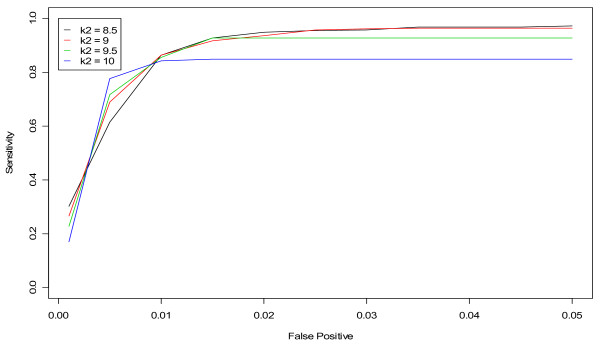
**ROC curves for detection of a simulated outbreak by day 3 in the non-flu seasons using various values of k in the MV CUSUM and applying the algorithm to six streams of data (unspecified infection and gastrointestinal from hospitals S, M, and L)**.

**Figure 9:**
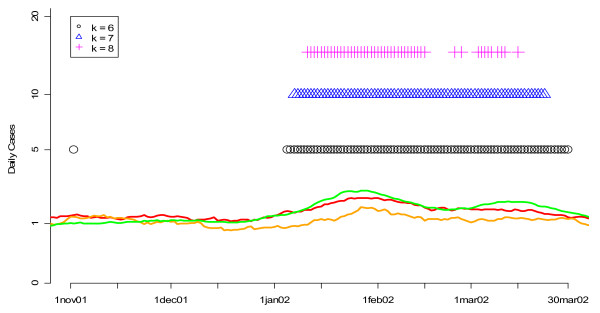
**Flags from the MV CUSUM algorithm for various values of k applied to three streams of unspecified infection data from hospitals S, M, and L during the winter of 2002**. Orange, red and green lines show the smoothed values for unspecified infection daily counts from hospitals S, M, and L, respectively. Flagging times are also represented along the time axis with vertical lines that are color coded to the symbol. Note that the vertical axis is on a square root scale because of its variance stabilizing properties.

**Figure 10:**
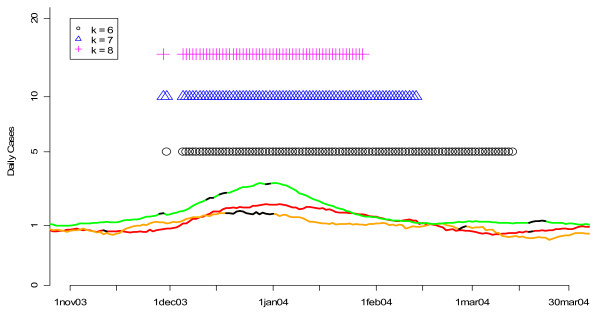
**Flags from the MV CUSUM algorithm for various values of k applied to three streams of unspecified infection data from hospitals S, M, and L during the winter of 2004**. Orange, red and green lines show the smoothed values for unspecified infection daily counts from hospitals S, M, and L, respectively. Black sections indicate missing data. Flagging times are also represented along the time axis with vertical lines that are color coded to the symbol. Note that the vertical axis is on a square root scale because of its variance stabilizing properties.

Finally, we applied our two-pronged fine-tuning approach to the *k *parameter in the MV CUSUM algorithm. We found that *k *is sensitive to the number of data streams on which the algorithm is applied. For example, when applied to three streams of data (daily standardized counts from hospitals S, M and L for unspecified infection or gastrointestinal), *k *= 7 was an optimal value (Figure 7). On the other hand, when applied to six streams of data (daily standardized counts from hospitals S, M and L for BOTH unspecified infection and gastrointestinal), *k *= 9 was optimal (Figure 8). When assessing the performance of the MV CUSUM algorithm for detecting "known" flu outbreaks in the winters of 2002 and 2004 for *k *= 6, 7, and 8 for three streams of data, we found that *k *= 6 had a slight advantage over *k *= 7 in the winter of 2002 while *k *= 8 did poorly at predicting the beginning of the flu outbreak (Figure 9). In the winter 2004, all values did similarly well (Figure 10). Given the optimality of *k *= 7 in the ROC approach, we selected *k *= 7 as the value for the MV CUSUM when applied to three streams of data in this data set.

As a final step in our fine-tuning analysis, we re-ran the analyses above on hospitals S, M, and L for unspecified infection and gastrointestinal data using the complete DC Department of Health dataset with daily counts from September 11, 2001 to June 19, 2006. The results for the univariate CUSUM and EXPO algorithms remained the same. However, the optimal parameter values for the MV CUSUM were different for the updated data. Two different values of *k *were now optimal for three streams of data. For three streams of gastrointestinal, *k *= 8 was optimal while for three streams of unspecified infection, *k *= 7 remained optimal. For six streams of data, *k *= 10 outperformed all other values of *k*. These results suggest that the MV CUSUM is sensitive to small changes in the data and thus, that fine-tuning the algorithm on an annual basis would be prudent.

We further studied the robustness of our results by rerunning the analyses above on three different hospitals for unspecified infection and respiratory daily counts. Again, the fine-tuned results for CUSUM and EXPO remained the same while the MV CUSUM optimal values for *k *changed. For three streams of unspecified infection, *k *= 5 was optimal and for three streams of respiratory, *k *= 6 was optimal.

Other studies have found a slight advantage to the MV CUSUM algorithm when applied to multiple streams of data [19]. We investigated whether this advantage continued after the algorithms' parameters had been fine-tuned. In order to assess this, we compared the fine-tuned versions of the CUSUM, EXPO, and MV CUSUM algorithms on three and six streams of data. When extending the univariate CUSUM and EXPO algorithms to three or six streams of data, the false positive rate of the algorithms applied to any one stream must be decreased to maintain a consistent daily false positive rate over multiple streams, i.e. the probability that one or more data streams will flag on a given day. Thus, using standard rules for multiple testing, we decrease the individual stream false positive rates to  and  when applying the algorithm to three and six streams of data, respectively and considered the extended versions of the algorithm to have flag if they flagged on any single stream with the reduced false positive rate.

**Figure 11:**
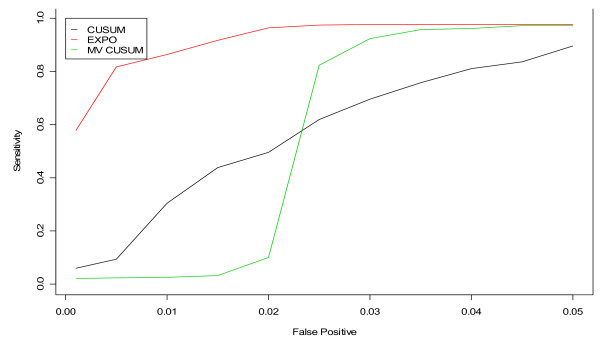
**ROC curves for detection of a simulated outbreak by day 3 in the non-flu seasons for CUSUM, EXPO, and MV CUSUM when applied to three streams of unspecified infection from hospitals S, M, and L**.

**Figure 12:**
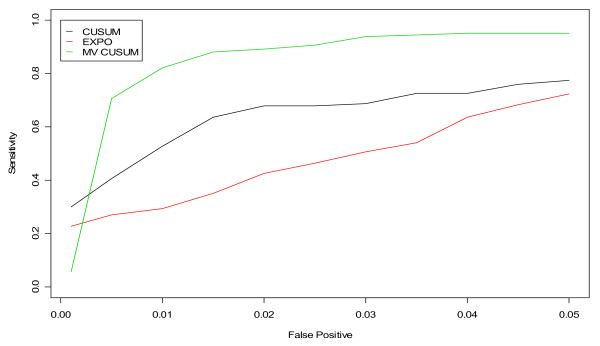
**ROC curves for detection of a simulated outbreak by day 3 in the non-flu seasons for CUSUM, EXPO, and MV CUSUM when applied to three streams of gastrointestinal data from hospitals S, M, and L**.

**Figure 13:**
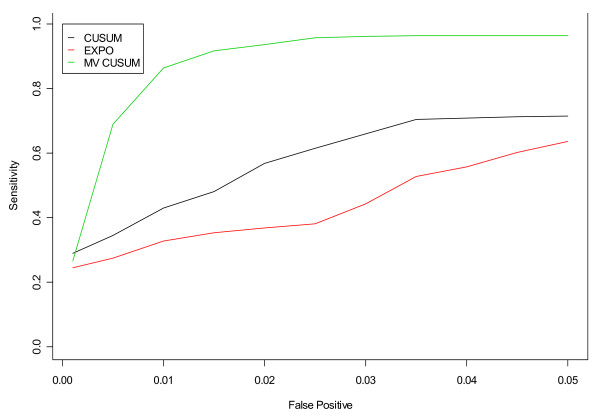
**ROC curves for detection of a simulated outbreak by day 3 in the non-flu seasons for CUSUM, EXPO, and MV CUSUM when applied to gastrointestinal and unspecified infection counts from hospitals S, M, and L**.

Figures 11 and 12 display the results of applying the fine-tuned algorithms to three streams of data, three steams of unspecified infection and three streams of gastrointestinal from hospitals S, M, and L, respectively. For unspecified infection (Figure 11), the application of EXPO to multiple data streams simultaneously outperformed both versions of the CUSUM. For gastrointestinal (Figure 12), the MV CUSUM outperformed the others. When applying the algorithms to six streams of data (e.g. unspecified infection + gastrointestinal data from all three hospitals), the MV CUSUM outperformed the EXPO and CUSUM algorithms (Figure 13).

**Figure 14:**
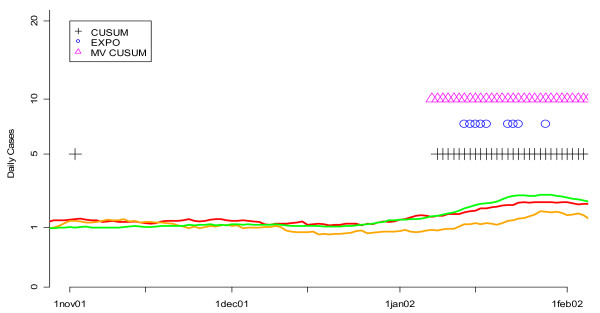
**Daily flags from the fine-tuned CUSUM, EXPO, and MV CUSUM algorithms when applied to three streams of unspecified infection data from hospitals S, M, and L during the winter of 2002**. Orange, red and green lines show the smoothed values for unspecified infection daily counts from hospitals S, M, and L, respectively. Flagging times are also represented along the time axis with vertical lines that are color coded to the symbol. Note that the vertical axis is on a square root scale because of its variance stabilizing properties.

**Figure 15:**
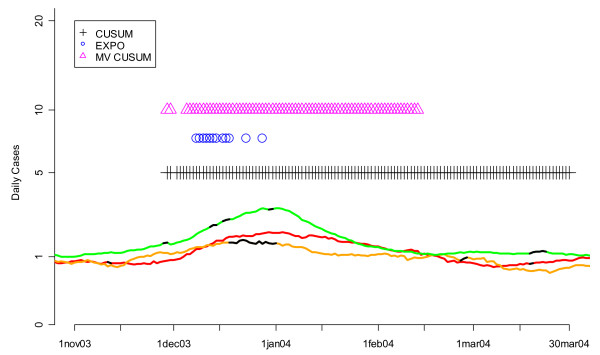
**Daily flags from the fine-tuned CUSUM, EXPO, and MV CUSUM algorithms when applied to three streams of unspecified infection data from hospitals S, M, and L during the winter of 2004**. Orange, red and green lines show the smoothed values for unspecified infection daily counts from hospitals S, M, and L, respectively. Black sections indicate missing data. Flagging times are also represented along the time axis with vertical lines that are color coded to the symbol. Note that the vertical axis is on a square root scale because of its variance stabilizing properties.

We also compared the three algorithms by checking them against the "known" flu outbreaks that occurred in DC during the winters of 2002 and 2004. Figure 14 demonstrates that the MV CUSUM outperformed EXPO at detecting the beginning of the flu outbreak in the winter of 2002. The same was true for the winter of 2004 (Figure 15). The MV CUSUM is the first algorithm to flag the beginning of the flu outbreak in both years. Contrary to the results from the ROC curve approach, EXPO did poorly at flagging the beginning of the flu outbreak. Taken together, these results strongly suggest that there is an advantage to using MV CUSUM to detect the beginning of the flu outbreak.

### Analysis of DC influenza data

**Figure 16:**
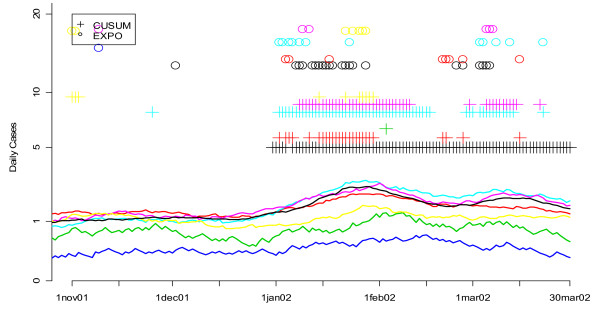
**Daily flags from the fine-tuned CUSUM and EXPO algorithms when applied to unspecified infection data from 7 hospitals during the winter of 2002**. Smoothed values for the number of unspecified infection cases are shown for seven hospitals, each with a different color curve. The flagging of the detection algorithms is represented by symbols (+ for CUSUM, o for EXPO) plotted according to the day they flagged on the horizontal axis and along different fixed values on the vertical axis to help distinguish more clearly between the algorithms being compared.

**Figure 17:**
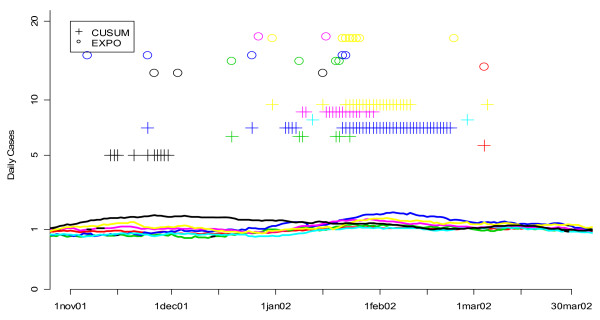
**Daily flags from the fine-tuned CUSUM and EXPO algorithms when applied to respiratory infection data from 7 hospitals during the winter of 2002**. Smoothed values for the number of respiratory cases are shown for seven hospitals, each with a different color curve. The flagging of the detection algorithms is represented by symbols (+ for CUSUM, o for EXPO) plotted according to the day they flagged on the horizontal axis and along different fixed values on the vertical axis to help distinguish more clearly between the algorithms being compared.

Our previous research suggested that of the eight syndrome groups available in the DC ERSSS, "unspecified infection" was most sensitive to determining the onset of the seasonal influenza outbreak in Washington DC. This was confirmed through a series of new analyses of the sort illustrated in Figures 16 and 17. In these graphs, smoothed values for the number of unspecified infection (Figure 16) and respiratory complaints (Figure 17) are shown for multiple hospitals. The flagging of the univariate detection algorithms is represented by symbols (+ for CUSUM, o for EXPO) plotted according to the day they flagged on the horizontal axis and along different fixed values on the vertical axis to help distinguish more clearly between the algorithms being compared. Figure 16 shows that the number of unspecified infection cases in most of the hospitals rises gradually through January, and both algorithms begin to flag, particularly the CUSUM, and do so consistently, starting early in January. On the other hand respiratory symptoms, shown for seven hospitals in Figure 17, do not exhibit such a strong increase and the univariate algorithms do not flag consistently. Indeed, some of the hospitals flag sporadically in November and December, arguably before the influenza outbreak began in DC that year. The other symptom groups available in the ERSSS exhibited even less of a consistent signal for seasonal influenza than respiratory symptoms.

**Figure 18:**
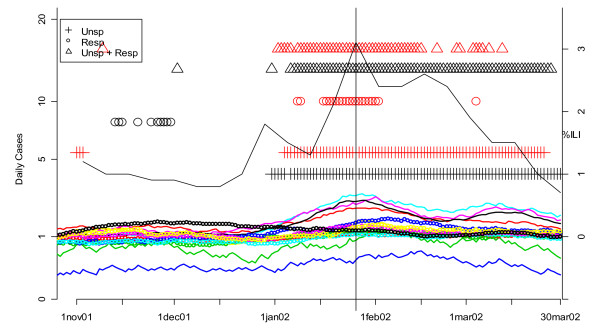
**Daily flags from potential Children's Hospital surveillance systems (black symbols) vs. all other hospitals (red symbols) plotted along with CDC sentinel physician data for winter 2002**. The smoothed number of cases is shown in the solid (unspecified infection) and dotted lines (respiratory complaints) and color-coded by hospital. The flagging of the detection algorithms is represented by black (for CNMC) and red (all other hospitals) symbols (+ for unspecified infection, o for respiratory, and Δ for MV unspecified infection and respiratory). The percentage of cases seen by sentinel physicians each week who have ILI is plotted on the right-hand vertical axis. The vertical line corresponds to the first date on which the sentinel physician data exceeds 2.5%, the CDC threshold on the national level for declaring the flu season.

Our previous analyses also suggested that analysis of unspecified infection counts from CNMC as a more sensitive indicator of the beginning of influenza outbreaks than unspecified infection data from other hospitals [13]. To confirm this, we compared the performance of the univariate and multivariate detection algorithms on unspecified infection and/or respiratory data from CNMC with a MV CUSUM analysis of unspecified and respiratory cases at the other six hospitals in DC's ERSSS. The results for winter 2002 are shown in Figure 18. The smoothed number of cases of both symptom groups for all seven hospitals is shown in the solid and dotted color-coded lines. The flagging of the detection algorithms is represented by black (for CNMC) and red (all other hospitals) symbols (+ for unspecified infection, o for respiratory, and Δ for MV unspecified infection and respiratory). As in Figures 8 and 9, the unspecified infection cases (+) flag earlier and more consistently than the respiratory cases (o). In addition, unspecified infection plus respiratory data (Δ) is not consistently more effective than unspecified infection alone (+). Whatever symptoms groups are analyzed however, the CNMC data (black symbols) flag before the comparable data from the other six hospitals (red symbols).

In order to evaluate the ERSSS data's ability to determine the beginning of the seasonal influenza outbreak in DC, we compared it to CDC's sentinel physician reports of influenza-like illness (ILI). The percentage of cases seen by sentinel physicians each week who have ILI is plotted on the right-hand vertical axis of Figure 18. The vertical line corresponds to the first date on which the sentinel physician data exceeds 2.5%. CDC uses this level as the threshold on the national level, but its application to one region is an approximation. In Figure 18 (2002), note that the local ERSSS data flag about two weeks before the CDC sentinel physician data reached the 2.5% threshold.

**Figure 19:**
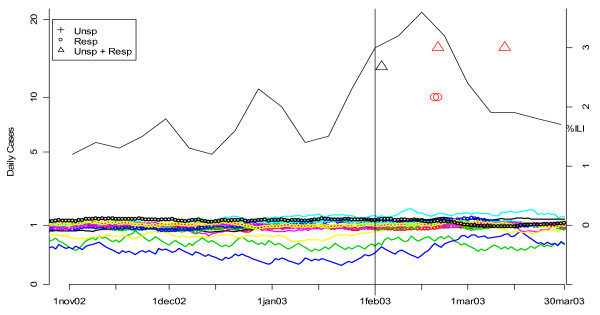
**Daily flags from potential Children's Hospital surveillance systems (black symbols) vs. all other hospitals (red symbols) plotted along with CDC sentinel physician data for winter 2003**. The smoothed number of cases is shown in the solid (unspecified infection) and dotted lines (respiratory complaints) and color-coded by hospital. The flagging of the detection algorithms is represented by black (for CNMC) and red (all other hospitals) symbols (+ for unspecified infection, o for respiratory, and Δ for MV unspecified infection and respiratory). The percentage of cases seen by sentinel physicians each week who have ILI is plotted on the right-hand vertical axis. The vertical line corresponds to the first date on which the sentinel physician data exceeds 2.5%, the CDC threshold on the national level for declaring the flu season.

**Figure 20:**
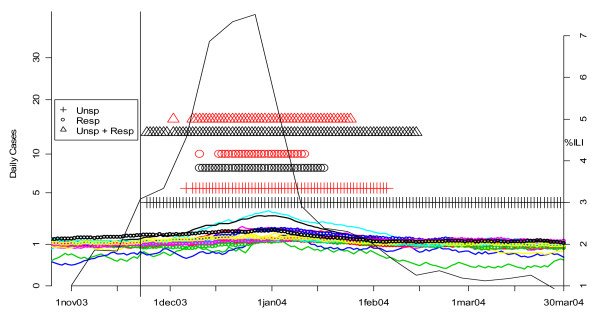
**Daily flags from potential Children's Hospital surveillance systems (black symbols) vs. all other hospitals (red symbols) plotted along with CDC sentinel physician data for winter 2004**. The smoothed number of cases is shown in the solid (unspecified infection) and dotted lines (respiratory complaints) and color-coded by hospital. The flagging of the detection algorithms is represented by black (for CNMC) and red (all other hospitals) symbols (+ for unspecified infection, o for respiratory, and Δ for MV unspecified infection and respiratory). The percentage of cases seen by sentinel physicians each week who have ILI is plotted on the right-hand vertical axis. The vertical line corresponds to the first date on which the sentinel physician data exceeds 2.5%, the CDC threshold on the national level for declaring the flu season.

**Figure 21:**
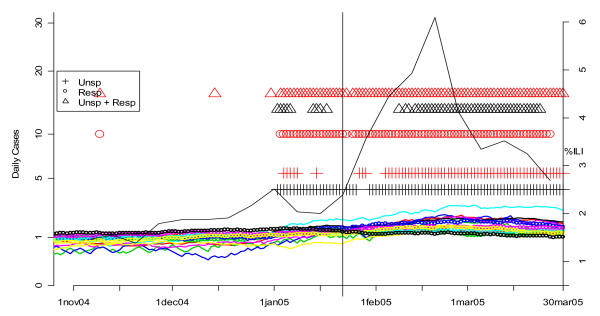
**Daily flags from potential Children's Hospital surveillance systems (black symbols) vs. all other hospitals (red symbols) plotted along with CDC sentinel physician data for winter 2005**. The smoothed number of cases is shown in the solid (unspecified infection) and dotted lines (respiratory complaints) and color-coded by hospital. The flagging of the detection algorithms is represented by black (for CNMC) and red (all other hospitals) symbols (+ for unspecified infection, o for respiratory, and Δ for MV unspecified infection and respiratory). The percentage of cases seen by sentinel physicians each week who have ILI is plotted on the right-hand vertical axis. The vertical line corresponds to the first date on which the sentinel physician data exceeds 2.5%, the CDC threshold on the national level for declaring the flu season.

**Figure 22:**
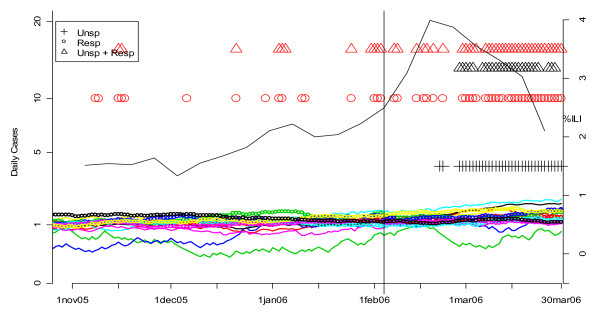
**Daily flags from potential Children's Hospital surveillance systems (black symbols) vs. all other hospitals (red symbols) plotted along with CDC sentinel physician data for winter 2006**. The smoothed number of cases is shown in the solid (unspecified infection) and dotted lines (respiratory complaints) and color-coded by hospital. The flagging of the detection algorithms is represented by black (for CNMC) and red (all other hospitals) symbols (+ for unspecified infection, o for respiratory, and Δ for MV unspecified infection and respiratory). The percentage of cases seen by sentinel physicians each week who have ILI is plotted on the right-hand vertical axis. The vertical line corresponds to the first date on which the sentinel physician data exceeds 2.5%, the CDC threshold on the national level for declaring the flu season.

Figures 19, 20, 21 and 22 present similar analyses for the winters of 2003, 2004, 2005, and 2006. Table 2 summarizes the results. The first row is based on an analysis of unspecified infection cases presenting at CNMC only, and the second row is based on a MV CUSUM analysis of unspecified infection cases at the other six hospitals in the analysis. The third row is based on the CDC's sentinel physician data. The date on which the first system alerted each year is indicated with bold, underlined type. In 2003, there was no clear flu outbreak in Washington and both the CNMC and other DC hospitals note this by not having any flags for the start of a flu season. In 2004, the ERSSS and CDC data flag at around the same time. The pattern for 2005 is similar to 2002, that is, the local ERSSS data have about a two week advantage. The data for 2006 are more ambiguous. The CDC sentinel physician systems flagged about two weeks before unspecified infection in CNMC in February, but the number of respiratory cases at the other hospitals was increasing through December and January, so it is difficult to say when the influenza outbreak actually started in DC that year. Indeed, the state epidemiologist's report to CDC fluctuated between "sporadic" and "local" from November 12 through May 20, with "no activity" reported for the week ending February 18, when the ERSSS and sentinel physician data were peaking [20].

**Table 2:** Date of first detection for Children's National Medical Center (CNMC) and six other DC hospitals and CDC's sentinel physician surveillance system (South Atlantic states) over four years with the first system to flag each year highlighted in bold and underlined and the number of days delay for the other systems shown below the date of flagging.

		*Year*			
***Surveillance system***	**2002**	**2003**	**2004**	**2005**	**2006**

CNMC	**Dec 31**	--	Nov 24 +2	**Jan 2**	Feb 27 +23
Other DC hospitals	Jan 4 +4	--	Dec 8 +14	Jan 4 +2	-
CDC sentinel physicians	Jan 26 +26	**Feb 1**	**Nov 22**	Jan 22 +20	**Feb 4**

It is interesting to note that in three of four years, CNMC consistently flags earlier (from 2 days up to 2 weeks earlier) than a multivariate analysis of all other DC hospitals. In 2006, the data for the other hospitals does not flag at all. These data suggest that the DC ERSSS, specifically unspecified infection data from CNMC, is adept at detecting the beginning of the outbreak in Washington.

## Discussion

Our results suggest that careful analysis of unspecified infection cases, and especially those from CNMC, was useful in detecting the beginning of influenza outbreaks in the District of Columbia. Comparing the earliest dates at which various systems flag, we found that for unspecified infection, CNMC consistently flags earlier than any of the other six hospitals. It alone also regularly flags before a multivariate analysis of the other six hospitals in our analysis. In addition, we found that although the respiratory syndrome group also provides information about influenza, it does not appear to add anything to the information contained in unspecified infection. These results suggest that the CNMC unspecified infection data be monitored more closely than others, and a flag in this system be regarded as having higher predictive value.

When we compared unspecified infection cases in CNMC and in the other DC hospitals (using optimal detection algorithms as discussed above) to CDC's sentinel physician data for the South Atlantic states for four years in which there was a discernable influenza outbreak in DC (2002, 2004, 2005, and 2006), we found that in two of these four years, syndromic surveillance from CNMC outperformed the other two systems, and in one year it flagged only two days after the CDC system. It is important to note that the CDC sentinel physician data system has a built in delay of 1-2 weeks, for example, data for patients seen in Week 12 in 2007 (March 18 - March 24) was published on March 31. Given this delay, analysis of the DC ERSSS provides a substantial advantage for real-time surveillance, particularly when using data from CNMC. Similar results were seen in an analysis of ER surveillance data from Boston [14] and New York [9], supporting the idea that children's symptoms are an especially sensitive indicator of the state of seasonal influenza.

These results are subject to limitations. Our method for setting the false positive rate assumes that there were no true outbreaks during the non-flu seasons. The cut-off value of five cases for the mean standardized daily counts in the non-flu seasons for the univariate CUSUM was developed after observing that 0.5 worked well for hospital C (used in the sensitivity analysis) which had a mean standardized count of 5.3 cases for respiratory infection while for hospital A with a mean standardized count of 4.8 for unspecified infection, 1.5 was superior. While not exact, this rule of thumb worked very well in the DC data and should only be used as a starting point for other data sets. We also acknowledge that we cannot generalize our results for the univariate CUSUM beyond a mean standardized count of 28.1 cases (e.g. the largest stream of daily counts available to us in the DC data). Similar caution must be taken when trying to extrapolate our results for the EXPO and MVCUSUM algorithms. In addition, the detection algorithms we used do not account for day of the week effects. According to our previous analyses [13], these do exist in the data but are smaller than in many other data sources. Adding day of the week terms to a prediction model or pre-filtering the data to remove day of the week effect would presumably result in a greater signal to noise ratio in the data, but not fundamentally change its nature. Thus increasing the sophistication of the detection algorithms by accounting for day of the week effects might improve their performance, but it is unlikely to alter the conclusions of this paper about the need for tuning the parameters. Furthermore, our simulation studies use a relatively simple linear pattern to describe the simulated outbreak. While actual outbreaks may have a very different pattern, the outbreaks of most public health concern, including seasonal and pandemic influenza, will grow in size over time, the general pattern that our simulations represent. More complex patterns might vary the specific results but again will not the key finding concerning the need to fine-tune parameters in statistical detection algorithms.

The primary limitation of the influenza analysis is that it is based on only four influenza outbreaks in one city, one of which was atypical. It should be replicated in other areas to confirm the potential benefit that careful monitoring of data from CNMC can have in a local area's syndromic surveillance system. The use of CDC sentinel surveillance data for the South Atlantic area, rather than for DC, is also a limitation. Unfortunately no more specific data of this type, or viral surveillance data, are available for the District.

## Conclusions

The results of this evaluation have implications for DC's ERSSS and for syndromic surveillance systems in general. First, the analysis shows that the choice of a detection algorithm's parameters matters in terms of statistical performance (here, assessed via sensitivity analysis and detection of known outbreaks). For the standard CUSUM algorithm, the optimal choice of *k *depended on the average number of cases per day outside the flu seasons. For the CUSUM based on deviations from an exponentially-weighted moving average (referred to as EXPO in this report), choosing k = 0.25 and *λ *= 0.20 was optimal, regardless of average number of cases. For the multivariate CUSUM, the choice of the *k *(defined differently than the *k *in the standard CUSUM) was much more sensitive to the data, and especially the number of data streams.

Although we explored only a limited set of detection algorithms and parameter values, our results suggest that simply using "canned" statistical detection algorithms with preset parameters may not give the optimal results for a given algorithm or data set. Rather, data need to be examined regularly using approaches similar to those above (perhaps with the consultation of a statistician) to fine-tune the parameters in the statistical detection algorithms to the data upon which they are being applied. Alternatively, Tokars and colleagues [21] explore how CDC's EARS algorithms can be improved by changing baseline periods and stratifying by day of the week.

The second conclusion is that, comparing the three detection algorithms applied to three or six data streams simultaneously in simulation studies, the multivariate CUSUM algorithm out-performed the two univariate methods in two of three settings examined in the simulation study, and in the detection of the beginning of the flu outbreak when applied to real data. The EXPO algorithm performed the best in one setting, namely detecting linearly increasing outbreaks in unspecified infection data from three hospitals. These results were also found in [19]. The results show that it is difficult to identify any particular characteristics of the data that would suggest that one algorithm is uniformly better than another. A system could be "tuned" to for different outbreak types, but that would require advance knowledge of what next season's flu would look like, which is clearly not available.

The optimal parameters and detection algorithms that we found in this analysis, of course, apply only to the particular data sets analyzed and the types of outbreaks studied (namely, linearly increasing outbreaks). The results cannot be generalized to other data streams and especially to other health departments. However, the conclusion that the parameters need to be tuned to the data does apply in general. Fine-tuning parameters on an annual or biannual basis would be prudent for any Emergency Room Syndromic Surveillance System.

In comparison with limitations of the ability of syndromic surveillance to detect bioterrorist events in a timely way unless they are very large and thus obvious [22], the analysis in this paper suggests that the most important contribution of syndromic surveillance to public health practice may be for natural disease outbreaks, such as seasonal and pandemic flu. The "situational awareness" that these systems provides can be quite valuable [23]. Indeed, in August 2009, the U.S. President's Council of Advisors on Science and Technology recommended that CDC aggregate real-time data from existing emergency department syndromic surveillance systems as a way to monitor the development and spread of the 2009-H1N1 influenza virus [24]. In addition, the World Health Organization now suggests monitoring outpatient or emergency department visits for acute respiratory illness, absenteeism rates from schools or work places, and other approaches that could be regarded as syndromic surveillance in countries in which 2009-H1N1 has already been established [25].

## Abbreviations

CDC: Centers for Disease Control and Prevention; CNMC: Children's National Medical Center; CUSUM: Cumulative summation detection algorithm; DC: District of Columbia; EARS: Early Aberrations Reporting System; ED: Emergency departments; ERSSS: Emergency Room Syndromic Surveillance System; EXPO: CUSUM algorithm based on deviations from an exponentially weighted moving average; ILI: Influenza-like illness; MV CUSUM: Multivariate CUSUM algorithm; ROC: Receiver operating characteristic curve; RSV: Respiratory syncytial virus.

## Competing interests

The authors declare that they have no competing interests.

## Authors' contributions

BAG, MAS, and AKJ: reviewed previous methods findings, designed analysis for this paper, conducted analysis, interpreted results, and drafted sections of the manuscript. JD-C: provided advice on the nature of the data, contributed to the design of the analysis and interpretation of the results. CG, GL, and SCW prepared data for analysis. All authors have read and approved the final version of the manuscript.

## Appendix

The following imputation strategy was used to impute missing data. Let X_t _represent the daily count for a given hospital and symptom group. In order to stabilize the variance, imputation was done in the square root scale, i.e. in terms of Z_t _= √X_t_. Define *μ *as the average value of Z_t _over all observed values and *σ *as the standard deviation of Z_t _over the same period. Similarly, define D_j _as the day of the week effect for day j (for Monday j = 1, and so on) which equals the average of Z_t _(for all days j) - *μ*. Finally, let e_t _be a random variable from a Normal distribution with mean 0 and standard deviation *σ*. For each missing value, we first calculate Y_t _as the exponentially weighted moving average based on the most recent observations of Z_t_. Transforming back to the original scale, the imputed value for day t is:

## Pre-publication history

The pre-publication history for this paper can be accessed here:

http://www.biomedcentral.com/1471-2458/9/483/prepub
